# Usefulness of CHA_2_DS_2_-VASc Scoring Systems for Predicting Risk of Perioperative Embolism in Patients of Cardiac Myxomas Underwent Surgical Treatment

**DOI:** 10.1038/srep39323

**Published:** 2016-12-16

**Authors:** Liang Yin, Jing Wang, Wei Li, Xinyu Ling, Qian Xue, Yufeng Zhang, Zhinong Wang

**Affiliations:** 1 Department of Cardiothoracic Surgery, Changzheng Hospital, the Second Military Medical University, Shanghai, China

## Abstract

Cardiac myxomas are rare but manifested with risk of embolism and often cause unexpected symptoms or sudden death. We retrospectively collected the medical records of patients diagnosed of cardiac myxomas at the cardiac center of our university. Overall 465 patients were included in this study, patients in the embolism group had significantly higher CHA_2_DS_2_-VASc scores (*P* = 0.005). In embolic group, stroke was recorded in 110 (77.14%) patients, while embolic events in the limbs were observed in 10 (2.15%) and 9(1.93%) developed splenic infarction. Patients in embolism group had older age (*P* = 0.021) and higher BMI (*P * <0.001) than those in non-embolism group. There was no significant difference between two groups in terms of time of mechanical ventilation (*P* = 0.065), ICU stay (*P* = 0.053), hospital stay (*P* = 0.071) and volume of drainage (*P* = 0.083), blood transfusions (*P* = 0.060) except that patients with embolic events had significantly higher incidence of postoperative atrial fibrillation (*P* = 0.032) and lower survival rate (P < 0.001). Furthermore, the CHA_2_DS_2_-VASc score was a significant predictor of embolism in patients with cardiac myxomas (*P* = 0.015; *P* = 0.003) and the Kaplan-Meier analysis obtained a higher rate of embolism in patients with higher stratification of CHA_2_DS_2_-VASc scores (*P* = 0.002). In conclusion, CHA_2_DS_2_-VASc scoring scheme was strongly predictive of stroke and embolic events in patients with cardiac myxomas.

Cardiac myxomas are rare compared with other cardiac diseases and tumors from other organs, with the incidence of approximately 0.3% in patients underwent cardiac surgery^1^,^2^,^3^,^4^. Cardiac myxomas are the mostly encountered benign cardiac tumors in surgery, with its incidence ranged from 70–80% in cardiac tumors^5^,^6^. Because of their various presentations, symptoms of cardiac myxomas vary greatly and they usually present themselves insidiously. Symptoms generated from cardiac tumors include arrhythmias, blood flow obstruction, embolization and other individual findings^7^,^8^,^9^. Prompt removal of these tumors has been established as the only mode of treatment for these patients^10^.

Type, size, and mobility of cardiac myxomas determine their clinical symptoms, which also greatly affect the incidence of embolization in patients. Embolism, intracardiac obstruction, and constitutional symptoms are mostly presented in patients, and the incidence of embolization was reported to be 20–40%^2^,^10^,^11^. Patients who occurred embolic events in the perioperative period had significantly higher morbidity and mortality, which may result in longer hospital stay and increased hospital costs and life quality was severely influenced following surgery^2^,^11^,^12^. Non-invasive procedure, such as echocardiography, computed tomography (CT), and magnetic resonance imaging (MRI) of the heart, are commonly used to diagnose cardiac myxomas and evaluate the risk of stroke in patients at risk^13^, allowing preoperative diagnosis with fair degree of accuracy. But only several scattered indicators and lack of systematic scoring system for predicting risk of stroke deter the assessing process preoperatively, and increased perioperative morbidity and long-term mortality may occur in concerned patients.

Originally, the CHA_2_DS_2_-VASc [congestive heart failure, hypertension, age ≥75 (doubled), diabetes, stroke (doubled), vascular disease, age 65–74, and sex category (female)] recommended by European Society of Cardiology (ESC) is an easy-to-remember means of assessing stroke risk of patients with AF, which is based on a point system in which 2 points are assigned for a history of stroke or TIA, or age ≥75; and 1 point each is assigned for age 65–74 years, a history of hypertension, diabetes, recent heart failure, vascular disease (myocardial infarction, complex aortic plaque, and peripheral arterial disease (PAD), including prior revascularization, amputation due to PAD, or angiographic evidence of PAD, etc.), and female sex^14^,^15^,^16^.

The CHA_2_DS_2_-VASc score can provide easy and effective assessment of patients with AF in which circumstances they can occur stroke or other thrombus-embolism events^17^. There was limited study having investigated the association between CHA_2_DS_2_-VASc score and prediction of risk of stroke or thrombus-embolism events in patients with cardiac myxomas who is also at high risk of embolism occurrence. In this study, we sought to explore the potential association between CHA_2_DS_2_-VASc score scheme and its utility of assessment for predicting embolic events in patients with cardiac myxomas underwent surgical treatment.

## Methods

### Patient Selection

Informed consent was obtained by each patient included in our study, and the consent procedure of our study was approved by Ethics Committee of Shanghai Changzheng Hospital, all methods performed in this study were carried out in accordance with relevant guidelines and regulations and all experimental protocols were approved by Ethics Committee of Shanghai Changzheng Hospital. This study included a series of patients who were underwent cardiac surgical treatment at the cardiothoracic center of the Second Military Medical University of PLA from January 2003 to December 2013. All patients with the diagnosis of cardiac myxomas treated with open heart surgery have been included in this retrospective study. Included patients were carried out surgery of tumor resection to treat cardiac myxomas with or without valve surgeries and other concomitant cardiac surgeries. Patients with history of recent myocardial infarction (MI) (less than one month), deep venous thrombus (DVT), preoperative renal or hepatic failure, infective endocarditis were excluded^3^,^18^.

### Patient Management

Complete medical records and follow-up data of each patient, including the medical history, neurologic examination, operative reports, pathology reports, CT or MRI of the brain, were collected using the patient database of our center, and follow-up of patients were conducted by contacting patients using telephone or by outpatient clinics. Pathological diagnosis of cardiac myxomas was obtained from the clinical pathological institute of our center. All patients presented with chest pain or older than 45 were carried out coronary angiography to rule out coronary heart disease (CHD)^19^. Preoperative management, anesthetic, and surgical techniques for all patients were standardized. Patients were transferred to the intensive care unit of our department postoperatively and managed accordingly.

### CHA_2_DS_2_-VASc score

The CHA_2_DS_2_-VASc score was calculated for all the patients by assigning 2 points for age ≥75 and for history of stroke, TIA, or thromboembolism and 1 point is assigned for congestive heart failure, hypertension, diabetes mellitus, age 65~75 years, vascular disease (myocardial infarction, complex aortic plaque, and PAD, including prior revascularization, amputation due to PAD, or angiographic evidence of PAD, etc.), and female sex category^20^,^21^.

### Endpoints and definitions

The main outcome endpoint of interest was new-onset ischemic stroke or any embolic event occurring during the perioperative period (before surgery during hospital, 30-day postoperative period or before discharge). Stroke was defined as new structural changes detected by CT or MRI imaging^22^, in patients with neurologic deficit lasting >24 hours without new structural changes in the brain by imaging, and the diagnosis of stroke was confirmed by a neurologist to minimize the error that may contribute to the bias generated in our study^2^,^3^,^23^,^24^. Embolic event occurring in other parts of the body except the brain was also detected in our study, which was included by presenting symptoms of embolism, analysis of the variables related to embolic potential and stroke lesion patterns, and finally confirmed by ultrasound or digital subtraction angiography (DSA)^10^,^25^.

### Statistical analysis

Statistical analysis was carried out using SPSS 18.0 software. Quantitative data are expressed as mean ± SD and were compared with 2-sample *t* tests for independent samples, whereas dichotomous variables were reported as absolute values and proportions. Differences in proportion were compared with a χ^2^ test or Fisher exact test as appropriate. Survival rates were estimated using the Kaplan–Meier Method. Variables significantly associated with occurrence of embolic event after univariate analysis (*P* < 0.05) were entered in a multivariable logistic regression model to identify the independent predictors of embolic event^26^. Additionally, we calculated the area under receiver operating characteristic (ROC) curve to assess the predictive value of CHA_2_DS_2_-VASc scores for risk of stroke in patients with myxomas^5^,^27^.

## Results

### Clinical Characteristics of patients

We collected data of patient’s age, gender, medical history and perioperative data. A total of 465 patients diagnosed of cardiac myxomas were included in this study, of these 152 were male patients and 313 were females, with a mean age of 53.39 ± 16.37, 58.19 ± 6.37 respectively. A total of 143(30.75%) patients developed stroke or embolic events, and patients were divided into two groups according to whether stroke or embolic event occurred. The result of analysis showed that patients in the embolism group had older age (*P* = 0.021) and higher BMI (*P * < 0.001) than those in non-embolism group, and history of hypertension (*P* = 0.035) and coronary artery disease (*P* = 0.004) were significantly more recorded in embolism group. Furthermore, patients in the non-embolism group had a higher frequency medication history of warfarin(*P* = 0.023) and aspirin (P < 0.001) than patients in the embolism group. The detailed baseline characteristics of patients were depicted in Table 1.

### Distribution of cardiac myxomas

According to the result of analysis, 91% (n = 423) of all the cardiac myxomas were found in the atrium, with 78% (n = 362) in the left atrium (LA) and 6% (n = 28) in the right atrium (RA). Whereas, biatrial cardiac myxomas occurred in 25 (5.45%) patients, and there was 13 (2.42%) cases of myxomas with the tumor invading left ventricle (LV). Additionally, the results also showed that patients who developed embolic events had significantly bigger cardiac myxomas in size (6.01 ± 9.2 cm vs 4.21 ± 3.9 cm, P = 0.021), and atypical type of myxomas according to previous report (myxomas with soft consistency and very fine villous extensions, an irregular or villous surface) was also more often encountered in embolism group (P < 0.001).

### Occurrence of stroke and embolic events

Stroke or embolism related events occurred in 143 patients during the perioperative period, with the incidence rate of 30.75%. In patients who developed stroke or embolic events, female (124 (87%)) contributed to the most contribution and with the remaining male genders, the average age of patients occurred embolism related events was 59.40 ± 10.9. In terms of type of embolic events, stroke was recorded in 110 (77.14%) patients with most of the patients presented the signs of neurological symptoms related to acute ischemic stroke, while embolic events in the limbs were observed in 10 (2.15%) patients. We also detected 9 (1.93%) patients who developed splenic infarction because of falling of the fragment of the myxomas, which was verified by abdominal CT scan. In addition, pulmonary embolism and retinal artery occlusion was detected in 7 (1.50%) patients respectively.

### CHA_2_DS_2_-VASc score

According to the algorithm of the score scheme, every point was assigned to each component of CHA_2_DS_2_-VASc score system for every patient included. Data of CHA_2_DS_2_-VASc scores of each patient included in our study were presented in Table 1. The exact percentage of patients in each point was as follows: 0-(n = 3, 8.57%), 1-(n = 6, 8.82%), 2-(n = 11, 19.64%), 3-(n = 15, 20.55%), 4-(n = 16, 26.23%), 5-(n = 25, 41.67%), 6-(n = 28, 44.44%), 7-(n = 17, 62.96%), 8-(n = 13, 100%), 9-(n = 9, 100%). Patients who developed stroke or embolism related event in the perioperative period had significant higher CHA_2_DS_2_-VASc score than those without embolism (*P* = 0.005), and the univariate and multivariate regression analysis showed that the CHA_2_DS_2_-VASc score were significant predictors of incidence of stroke or embolic events in patients of myxoma (*P* = 0.015; *P* = 0.003) (Tables 2 and 3).

**Figure 1:**
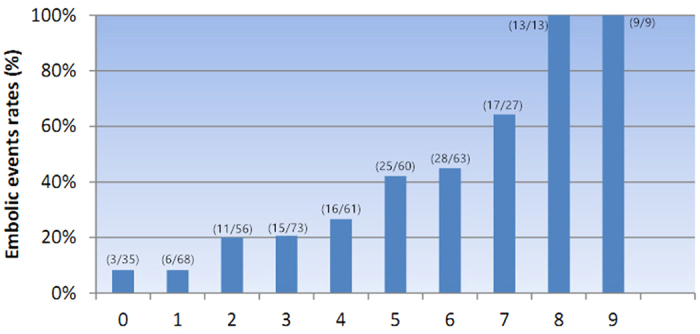
Embolic events rates and CHA_2_DS_2_-VASc scores. The embolic events rates incrementally increased as the CHA_2_DS_2_-VASc score increased.

As shown in Fig. 1, the incidence rate of stroke or embolic event was strongly associated with CHA_2_DS_2_-VASc score. The incidence of embolic events incrementally increased with CHA_2_DS_2_-VASc score raised, and higher score was predictive of higher stroke and embolism incidence rate.

**Figure 2:**
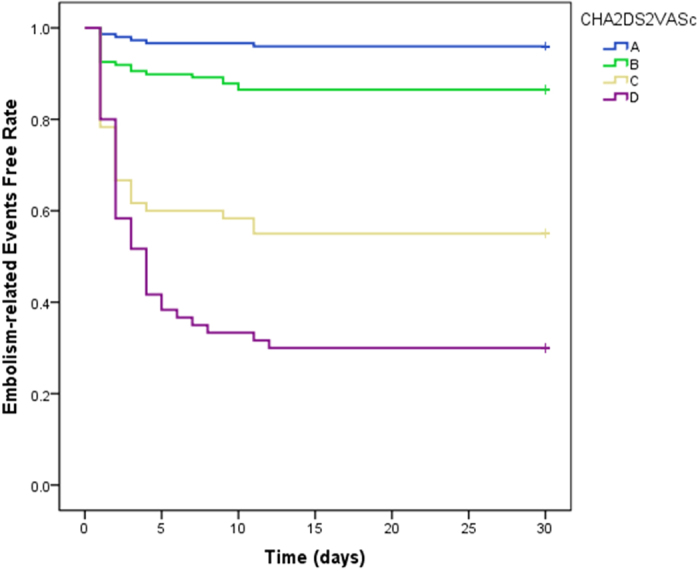
Embolism-related events free rate curves for patients with CHA_2_DS_2_-VASc score. The Kaplan-Meier survival analysis showed that patients with higher stratification of CHA_2_DS_2_-VASc score had a higher event rate than those who had lower stratification of CHA_2_DS_2_-VASc score. (P = 0.002). A-(0-1 point), B-(2–3 points), C-(4–5 points), D-(≥6 points).

In the Kaplan-Meier analysis, we stratified CHA_2_DS_2_-VASc scores. We categorized score 0–1 as A, score 2–3 as B, score 4–5 as C and score greater than 6 as D. As demonstrated in Fig. 2, the cumulative event rates of stroke and embolic events in Kaplan-Meier survival curves showed that patients with higher stratification of CHA_2_DS_2_-VASc score had a significant higher incidence rate of perioperative stroke or embolic events (*P* = 0.002).

**Figure 3:**
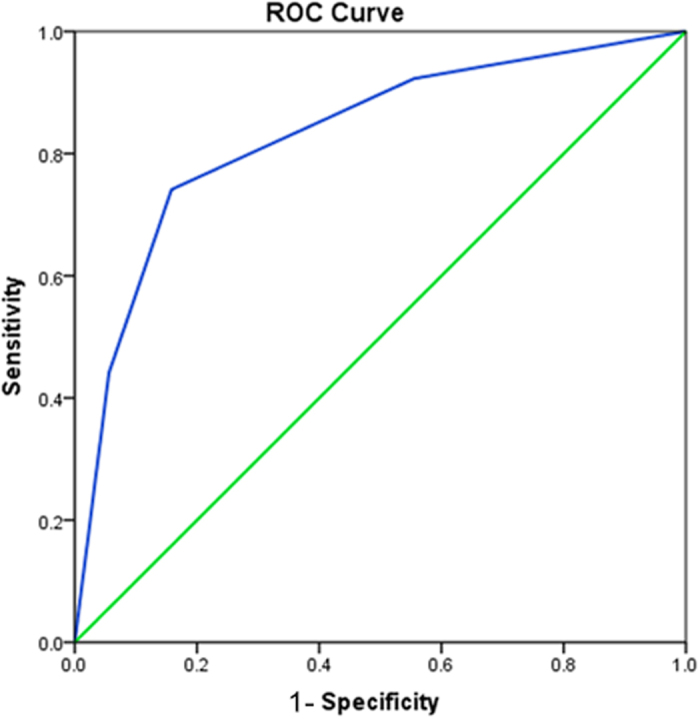
Predictive value of CHA_2_DS_2_-VASc score for incidence of stroke and embolic evens under ROC, area under ROC was 0.83 (95%CI, 0.789–0.873, p < 0.001).

The predictive value of CHA_2_DS_2_-VASc score for incidence of stroke and embolic event in patients diagnosed with cardiac myxomas was depicted in Fig. 3, the area under ROC was 0.83 (95%CI, 0.789–0.873, p < 0.001).

### Multivariable logistic analysis

The relationship of myxomas-related stroke or embolism with risk factors was assessed using the multivariate logistic regression analysis. After adjustment for history of anticoagulants use and history of atrial fibrillation, independently and significantly high risk factors contributing to stroke and embolism in patients with cardiac myxomas in our study were age (OR: 0.765; p = 0.021), type of myxomas (OR: 1.453; p = 0.001), size of myxomas (OR: 0.411; p = 0.014), E/e’ ratio (OR: 1.022 = 0.061), LAD (OR: 1.014; p = 0.031) and CHA_2_DS_2_-VASc score (OR: 0.243; p = 0.003) (Table 3). The result of analysis revealed thatCHA_2_DS_2_-VASc score was significantly higher in the embolism group, and higher CHA_2_DS_2_-VASc score was significant predictors of embolic events. Additionally, larger diameter of myxomas and LAD were also relatively predictors in patients with cardiac myxomas.

### In-hospital mortality and long-term follow up

Among 465 patients diagnosed with cardiac myxomas included in our study underwent surgical treatment at our institution, 5 (1.07%) in-hospital deaths (3 in embolism group and 2 no-embolism group) occurred (death occurring within 30 days of operation or before discharge) because of bleeding and serious heart failure. Complications related to surgery postoperatively were recorded in 63 (13.54%) patients, and there were 26 (5.59%) patients had episodes of arrhythmias and 27 (5.80%) had major bleeding, renal dysfunction and wound infection were both observed in 5 (1.07%), 8 (1.72%) case respectively.

Information on postoperative complications related to surgery was depicted in Table 4. There was no significant difference between two groups in terms of time of mechanical ventilation (*P* = 0.065), ICU stay (*P* = 0.053), hospital stay (*P* = 0.071) and volume of drainage (*P* = 0.081), blood transfusions (*P* = 0.060), as well as other perioperative variables except that patients with embolic events had significantly higher incidence of postoperative atrial fibrillation (*P* = 0.0325).

## Discussion

In this study, we present a series of cardiac myxomas involving reviewed comprehensive clinical data of these consecutively diagnosed patients in our center. Comparing with most of previous reports, we uniquely utilize the CHA_2_DS_2_-VASc score system, which used to assess the stroke risk of patients with AF, for predicting embolism related events in patients diagnosed of cardiac myxomas who were also at high risk of occurring such perioperative events.

In our data analysis, the patients included with cardiac myxomas contributed to 3.21% of all the continuous patients underwent cardiac surgeries in our center, which was similar to previous studies^4^,^19^,^28^. And a percentage of 30.75% of stroke or embolic events contributed to the overall included patients, a little slightly higher than the incidence reported in other studies which ranged from 20–25%^29^. We uniquely utilize the CHA_2_DS_2_-VASc score scheme, which used to be an easy-to-remember tool to identify patients with AF who are at risk of occurring stoke, to assess the risk of stroke and embolic event in patients diagnosed of cardiac myxomas. The result from our study showed that CHA_2_DS_2_-VASc score scheme was also a useful predictive system for predicting stroke and embolic events in patients with cardiac myxomas, and patients with CHA_2_DS_2_-VASc score at higher stratification had a significant higher occurrence of embolization related events than those who had lower stratification. We also calculated the individual occurrence of embolism event in each patient based on different points from naught to the highest whose result are shown in Fig. 1. Furthermore, in our multivariable regression analysis, the CHA_2_DS_2_-VASc score was valuable predictors for embolism in patients with cardiac myxomas.

With the incidence rate of 0.5 per million each year, cardiac myxomas can occur at any age stage of patients, while usually females with the age 40~60 are commonly encountered, and mostly located in the LA and RA, which are rarely seen in left and right ventricle^1^,^3^,^28^,^30^. Intracardiac obstruction and peripheral embolism are the two main constitutional symptoms in most patients diagnosed with cardiac myxomas. In early reports, a frequency of 30~40% of embolization have been reported^5^,^6^,^11^,^28^,^31^,^32^. Recent reports of the sign of stroke and embolism associated with cardiac myxomas showed a downward trend with the frequency of 20~25%^6^, and in this retrospective study we reported the incidence of 30.75%.

The CHA_2_DS_2_-VASc score scheme is accurate and is both easily and widely used in prediction of severity and outcome of strokes, providing decision-making management in patients with AF^17^,^18^. As demonstrated in previous studies, an incremental deterioration of left ventricle (LV) systolic and diastolic dysfunction may cause left atrium (LA) enlargement, which is strongly associated with the occurrence of postoperative atrial fibrillation (POAF) which is associated with stroke or embolism^16^,^21^, and whose underlying mechanism were similar to patients with myxomas. Congestive heart failure, one of the most important components in CHA_2_DS_2_-VASc score system, is morbid condition that shares common risk factors and frequently coexisted with cardiac myxomas, which ranged 46~47% in this study. Furthermore, the unstable hemodynamic status of patients with myxomas also contribute to deteriorated embolism events occurrence, which increased morbidity and mortality significantly^20^. Hypertension, diabetes, and vascular disease, another several components of CHA_2_DS_2_-VASc score system, also can promote the incidence of stoke and embolic event in patients with myxomas.

Although originally CHA_2_DS_2_-VASc score was not designed to identify patients with cardiac myxomas who were also at risk of occurring stroke and embolism, which was similar to patients of AF, and the underlying mechanism and potential under which embolic event occurred were either different between two groups of patients. The results from our study showed that CHA_2_DS_2_-VASc score scheme was also an effective predictive tool for identifying myxomas patients at risk of embolism. Additionally, patients with CHA_2_DS_2_-VASc score at higher stratification had a significant higher occurrence of embolization related events than those who had lower stratification. This relationship may be helpful to discriminate patients who were predisposed to high stroke and embolism occurrence, and which may also provide us decision-making strategies of preoperative prophylactic medication. We recommended that high risk patients diagnosed of cardiac myxomas with a CHA_2_DS_2_-VASc score greater than 4 may be strongly associated with the embolic event in the perioperative period, and preoperative prophylactic management may be needed in these patients to minimize the risk of embolism. In patients with higher CHA_2_DS_2_-VASc score, we recommended use of anticoagulants including low molecular weight heparin (LMWH), warfarin or new oral anticoagulant (NOAC) to minimize the risk of thrombosis. Additionally, owing to different mechanism from embolism caused by cardiovascular disease, unstable and atypical type of myxomas were also independent risk factors for embolism, so early accurate diagnose and typing of myxomas and surgical removal were critical in preventing embolism events occurred. In surgical techniques of removing myxomas, embolic excision of the tumor without residue including pedicles was essential, repeated syringe with saline inside the atrial and ventricular chambers on arrested heart was also critical.

In summary, cardiac myxomas comprise a small percentage of cardiac diseases which also had high risk of stroke and embolism. Using CHA_2_DS_2_-VASc score to identifying patients at risk of stroke and embolism was an effective management in the perioperative period. Once identified as patients at high risk with high CHA_2_DS_2_-VASc score, elaborative prophylactic management was necessary to decrease perioperative morbidity and mortality.

The main limitation of our study is its retrospective design. Also, we evaluated the risk factors consisting of CHA_2_DS_2_-VASc score alone without analysis with other risk factors which may contribute to bias in our study. And the follow-up period of patients were not investigated in this study. Although the meaningful findings in our study, CHA_2_DS_2_-VASc score is useful to predict embolic events in most populations including patients with AF, and this finding may not entail highly specific and sensitive predictions in patients with cardiac myxomas. Nevertheless, our study provided useful information related to the risk factors of stroke and embolism in patients with cardiac myxomas. A larger clinical trial is necessary to assess the underlying usefulness of CHA_2_DS_2_-VASc scores in predicting risk of stroke to assure whose clinical utility.

## Additional Information

**How to cite this article**: Yin, L. *et al*. Usefulness of CHA_2_DS_2_-VASc Scoring Systems for Predicting Risk of Perioperative Embolism in Patients of Cardiac Myxomas Underwent Surgical Treatment. *Sci. Rep.* **6**, 39323; doi: 10.1038/srep39323 (2016).

**Publisher’s note:** Springer Nature remains neutral with regard to jurisdictional claims in published maps and institutional affiliations.

## Figures and Tables

**Table 1:** Baseline characteristics of patients.

	Embolism (n = 143)	no-Embolism (n = 322)	*p* value
Age (y)	59.4 ± 10.9	52.3 ± 12.4	0.021
Age 65–75 y	47 (32.90%)	88 (27.3%)	0.045
Age ≥75 y	5 (3.50%)	6 (1.86%)	0.077
Female, n (%)	99 (69.23%)	214 (66.46%)	0.407
Smoke, n (%)	50 (35.2%)	135 (41.92%)	0.438
BMI (kg/m^2^)	26.1 ± 2.8	23.3 ± 2.6	0.001
Medical history, n (%)			
Heart failure	67 (46.85%)	154 (47.82%)	0.239
Hypertension	54 (37.76%)	78 (24.22%)	0.035
Diabetes mellitus	32 (22.37%)	49 (15.22%)	0.061
Prior stroke or TIA	5 (3.50%)	11 (3.41%)	0.965
Coronary artery disease	79 (55.24%)	32 (9.93%)	0.004
Carotid artery disease	45 (31.46%)	33 (23.07%)	0.127
Peripheral arterial disease	38 (26.57%)	26 (8.07%)	056
Atrial fibrillation	34 (23.77%)	79 (24.53%)	0.876
Atrial flutter	11 (7.69%)	35 (10.86%)	0.551
Concomitant surgery, n (%)			
MVP	13 (9.09%)	34 (10.55%)	0.411
MVR	19 (13.28%)	41 (12.73%)	0.901
AVR	11 (7.69%)	36 (11.18%)	0.658
TVP	8 (5.59%)	19 (5.90%)	0.284
TVR	0 (0%)	3 (0.93%)	0.077
CABG	2 (1.39%)	1 (0.31%)	0.056
Preoperative medication, n (%)			
Warfarin	4 (2.79%)	15 (4.65%)	0.023
Statins	7 (4.89%)	27 (8.38%)	0.047
Aspirin	6 (4.19%)	51 (15.83%)	<0.001
Clopidogrel	1 (0.69%)	9 (2.79%)	0.054
Myxomas			
Size (cm)	6.01 ± 9.2	4.21 ± 3.9	0.021
Atypical type*, n (%)	67 (46.85%)	78 (24.22%)	<0.0001
Distribution of cardiac myxomas			
LA	112 (78.32%)	250 (77.63%)	0.87
RA	10 (6.99%)	18 (5.59%)	0.55
Biatrial	11 (7.69%)	14 (4.35%)	0.14
LV	4 (2.79%)	9 (2.80%)	0.99
Echocardiographic			
LVEF (%)	57.7 ± 5.3	56.5 ± 4.2	0.656
LAD (cm)	6.1 ± 3.4	5.3 ± 1.2	0.011
E/e’ ratio	16.5 ± 3.2	11.2 ± 2.5	<0.0001
CHA2DS2-VASc score	3.4 ± 1.7	1.5 ± 1.3	0.005

Data are presented as n (%) or mean ± SD. BMI, body mass index; TIA, transient ischemic attack; MVP, mitral valvuloplasty.

MVR, mitral valve replacement; AVR, aortic valve replacement; TVP, tricuspid valvuloplasty; TVR, tricuspid valve replacement.

CABG, coronary artery bypass grafting; LA, left atrium; RA, right atrium; LV, left ventricle; LVEF, left ventricular ejection fraction.

*With an irregular or villous surface and a soft consistency.

**Table 2:** Univariate regression analysis for risk factors of embolism.

Variable	Odds ratio	95%CI	*p* value
Age (y)	0.965	0.907–1.026	0.254
Gender	0.901	0.807–1.001	0.321
Smoke	1.783	0.691–2.909	0.625
Diabetes	2.476	1.732–13.817	0.111
Hypertension	0.937	0.181–10.009	0.687
BMI (kg/m^2^)	6.623	1.117–9.204	<0.0001
Prior stroke or TIA	0.965	0.875–2.334	0.197
Size of myxomas	1.123	1.007–3.084	0.002
Atypical type	9.133	1.117–32.204	0.001
History of anticoagulation	1.003	0.945–2.204	0.065
LAD (cm)	1.694	1.084–2.807	0.022
E/e’ ratio	8.006	0.300–12.098	0.045
Arrhythmia	2.341	1.800–3.098	0.068
CHA_2_DS_2_–VASc score	1.009	0.897–2.007	0.015

CI, Confidence interval; BMI, body mass index; LAD, left atrial diameter.

**Table 3:** Multivariate regression analysis for risk factors of embolism.

Variable	Odds ratio	95%CI	*p* value
Age (y)	0.765	0.507–1.626	0.021
BMI (kg/m2)	0.237	0.112–0.894	0.001
E/e’ ratio	1.022	0.976–1.724	0.061
Size of myxomas	0.411	0.124–1.081	0.014
Atypical type	1.453	1.004–5.984	0.001
LAD (cm)	1.014	0.651–1.147	0.031
CHA2DS2–VASc score	0.243	0.103–0.844	0.003

CI, Confidence interval; BMI, body mass index; LAD, left atrial diameter.

**Table 4:** Perioperative data of patients.

	Embolism (n = 143)	no-Embolism (n = 322)	p
Operation time (min)	131.55 ± 38.95	135.00 ± 21.64	0.198
Drainage (ml)	450.43 ± 346.40	567.66 ± 556.00	0.083
Mechanical ventilation time (d)	2.34 ± 3.68	2.12 ± 0.62	0.065
Transfusion (ml)	321.22 ± 657.29	292.66 ± 567.18	0.060
ICU stay (d)	1.63 ± 0.88	1.48 ± 1.00	0.053
Total hospital stay (d)	12.39 ± 4.67	11.65 ± 3.69	0.071
Wound infection, n (%)	3 (2.09)	5 (1.55)	0.625
Renal failure, n (%)	1 (0.21)	4 (1.24)	0.067
POAF	18 (12.58)	8 (2.48)	0.032
Major bleeding	10 (6.99)	17 (5.27)	0.052
In-hospital deaths, n (%)	3 (2.09)	2 (0.62)	0.059

ICU, Intensive Care Unit, POAF, postoperative atrial fibrillation.
